# Index of cardiac electrophysiological balance in patients with vasovagal syncope confirmed by head-up tilt test

**DOI:** 10.3389/fcvm.2026.1743842

**Published:** 2026-03-17

**Authors:** Lifei Tian, Hong Hou, Le Zhang, Yan Gao

**Affiliations:** 1 Department of General Surgery, Shaanxi Provincial People’s Hospital, Xi’an, China; 2 Department of Cardiac Function, Xi’an Third Hospital/Affiliated Hospital of Northwest University, Xi’an, China; 3 Department of Cardiac Function, Shaanxi Provincial People’s Hospital, Xi’an, China

**Keywords:** autonomic nervous system, ECG parameters, head-up tilt test, index of cardiac electrophysiological balance, vasovagal syncope

## Abstract

**Background:**

Vasovagal Syncope (VVS) is a transient loss of consciousness caused by reflex vasodilatation and/or bradycardia, typically triggered by orthostatic stress, emotion, or pain. It is the most common form of neurally mediated syncope, carries a benign prognosis, yet recurrent episodes impair quality of life and require risk stratification and tailored management. The Index of Cardiac Electrophysiological Balance (ICEB) is a novel index designed to quantify the ventricular depolarization—repolarization electrophysiological balance and exhibits a bidirectional relationship with autonomic nervous function. In this study, we investigated the changes in ICEB among patients with VVS.

**Methods:**

The clinical data of 266 patients with VVS, confirmed by Head-up tilt test (HUTT) at Shaanxi Provincial People's Hospital from August 2021 to February 2025 were retrospectively collected as study group. A total of 201 healthy individuals who completed routine physical examinations during the same period were enrolled as the control group. Electrocardiogram (ECG) parameters (RR, P, QRS, QT) and ICEB were calculated and statistical analyzed during the HUTT in 5-minute supine phase or at rest.

**Results:**

The QT interval, corrected QT (QTc) interval, ICEB and corrected ICEB (ICEBc) were lower (*P* < 0.001, respectively) in VVS group than in control group, with Type 2 showing the lowest level, even lower than those in Type 1 and Type 3. Pearson's correlation coefficient indicated that there was no significant correlation between the shortened cardiac electrophysiological parameters and asystole duration in Type 2. After adjusting for age, sex, body mass index, systolic blood pressure and diastolic blood pressure, VVS patients still had significantly lower ICEB, ICEBc compared with the control group.

**Conclusion:**

In our study, we tried to demonstrate the relationship between ICEB and VVS confirmed by HUTT. We indicate that ICEB and ICEBc were shortened in patients with VVS at baseline, which may be attributed to autonomic dysfunction.

## 1 Introduction

The Index of Cardiac Electrophysiological Balance (ICEB) has gained increasing attention as an emerging electrophysiological indicator, which associates with the imbalance of autonomic nervous system (ANS) (1, 2). Calculated as the ratio of the QT interval to the QRS duration on the electrocardiogram, ICEB more accurately reflects the balance of cardiac electrical activity compared to traditional QT or QTc intervals. Studies have reported that ICEB was associated with several diseases or medical conditions, including Long QT Syndrome (LQTS), coronary slow flow and myocardial bridge, and may be a measurable marker of Torsades de Pointes (TdP) or non-TdP mediated ventricular arrhythmias (3–5). Neurally mediated reflex syncope-commonly termed Vasovagal Syncope (VVS)-denotes a sudden, transient failure of autonomic cardiovascular control, usually triggered by orthostatic stress, emotion or pain. The ensuing hypotension and/or bradycardia causes transient global cerebral hypoperfusion, producing a brief loss of consciousness that resolves rapidly and without residual neurologic deficit. Head-up tilt test (HUTT) is a commonly used diagnostic tool for syncope in clinical practice, especially in suspected VVS (6). It has become increasingly popular among physiologists and physicians due to its high sensitivity and specificity since its first application for the diagnosis of syncope in 1986 (7). The latest guidelines from the American College of Cardiology/American Heart Association/Heart Rhythm Society (ACC/AHA/HRS) and the European Society of Cardiology (ESC) both recommend HUTT as a Class IIa indication in the diagnosis of syncope (8, 9). HUTT reproduces patient's symptoms, including prodrome and the loss of consciousness, evaluates haemodynamic responses in ANS by changing position. Positive HUTT patients demonstrate excessive excitation of the vagus nerve, which often manifested as- abnormal heart rate variability (HRV) parameters previously (10), leading to sudden drops in heart rate and blood pressure. However, no one has reported any distinctions in ICEB, which reflects both the state of autonomic nervous function and the balance of ventricular depolarization and repolarization, among patients with VVS. In this study, we investigated the changes in ICEB among patients with VVS.

## 2 Materials and methods

### 2.1 Participants

**Figure 1:**
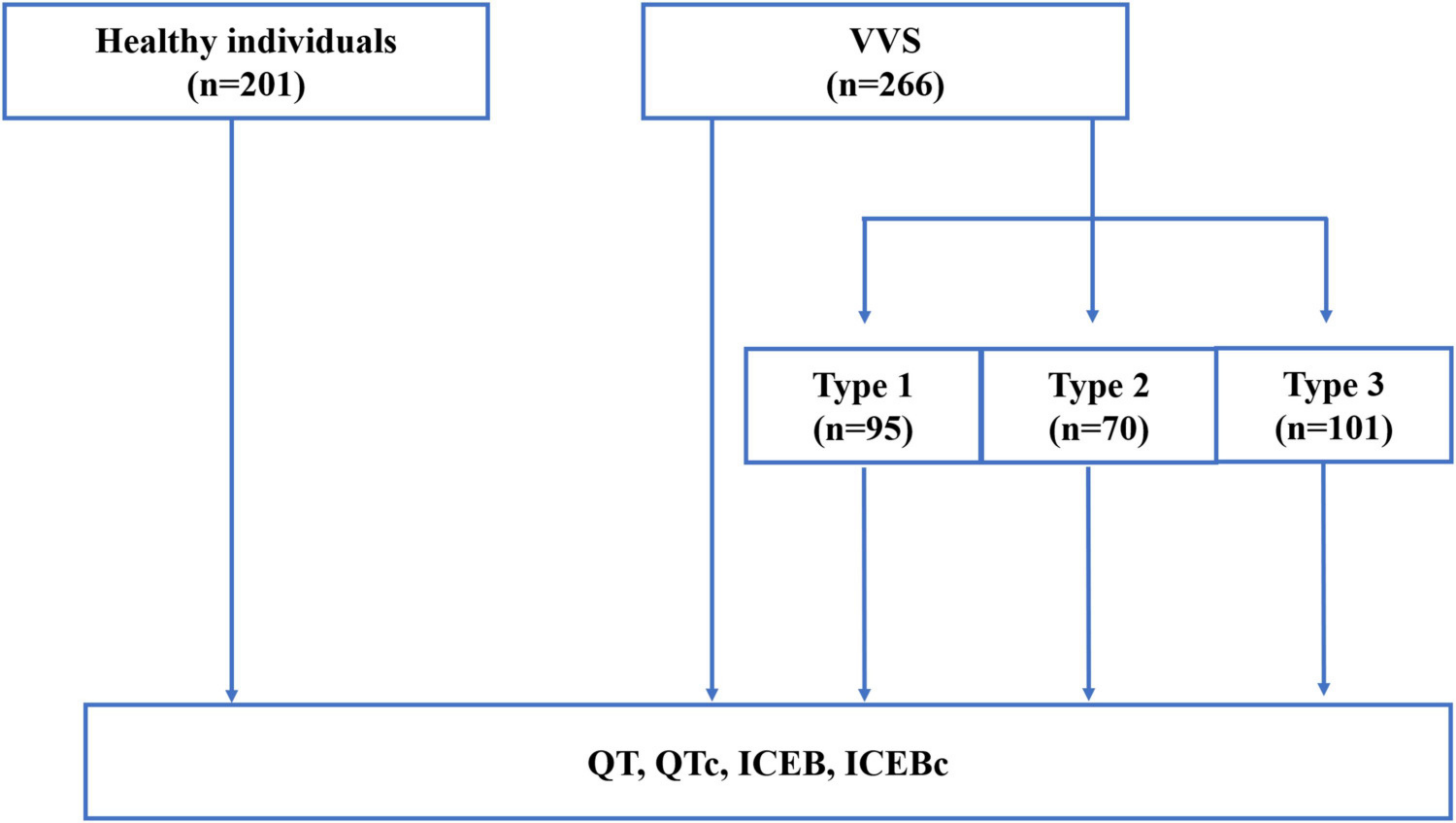
Flow chart of the study. VVS, Vasovagal Syncope; ICEB, The Index of Cardiac Electrophysiological Balance; ICEBc, corrected ICEB.

The clinical data of 266 patients with positive HUTT results at Shaanxi Provincial People's Hospital from August 2021 to February 2025 were retrospectively collected as study group. All patients had sinus rhythm. Patients with coronary heart disease, cardiomyopathy, hypertension, atrioventricular block, long/short QT syndrome, diabetes mellitus and other endocrine diseases were excluded. No patient was receiving any medication known to prolong the QT interval (e.g., tricyclic antidepressants, class I or III antiarrhythmics, fluoroquinolone antibiotics, or other drugs with established effect potential). A control group consisting of 201 healthy individuals who completed routine physical examinations during the same period was established for comparison. Inclusion criteria: (1) Absence of syncope or unexplained transient loss of consciousness; (2) Normal physical examination, resting 12-lead ECG, postero-anterior chest radiograph, and comprehensive transthoracic echocardiography; (3) No evidence of chronic systemic disease (hypertension, diabetes mellitus, coronary artery disease, cerebrovascular disease, hepatic or renal insufficiency); (4) No use of any medication with autonomic or direct cardiovascular activity (e.g., β-adrenergic antagonists, antidepressants, antiepileptic drugs, anti-arrhythmic agents). Exclusion criteria: (1) Any ECG abnormality (e.g., arrhythmia, significant ST-T changes, prolonged/shortened QTc, Brugada pattern, pre-excitation, conduction block); (2) Severe neurological or psychiatric disorders, including: Parkinson's disease, dementia, major anxiety/depression, schizophrenia; (3) Metabolic or endocrine diseases: diabetes mellitus, hyperthyroidism, adrenal disorders, electrolyte disturbances. The study was approved by the local ethics committee of the Shaanxi Provincial People's Hospital. (Approval ID: 2025K-S186) The flow chart of this study is shown in Figure 1.

#### 2.1.1 Sample-size justification

An *a priori* power analysis was performed with G*Power 3.1.9.7 for an independent two-tailed t-test. With *α* = 0.05 and power = 0.90, the minimum sample size was 86 subjects per group. Thus, the final sample size (201 controls/266 patients) exceeds the required number and is sufficient to detect the prespecified effect.

### 2.2 Electrocardiographic parameters

12-lead ECG was obtained during the HUTT in 5-minute supine phase in study group. In control group, 12-lead ECG was obtained by ECG-3350p (Shanghai Photoelectric Technology Co., Ltd.). Baseline wander was removed using a 0.5 Hz high-pass filter; 50 Hz power-line interference was eliminated with the device's built-in notch filter; high-frequency myoelectric noise was reduced via the device's dedicated myoelectric filter. Two independent, board-certified cardiologists (with >5 years of experience in cardiac electrophysiology) blinded to group assignment manually measured ECG parameters on lead V5 (selected for its robust representation of ventricular depolarization/repolarization in sinus rhythm, per clinical practice guidelines). Discrepancies between observers were resolved by consensus with a third senior cardiologist. Measured parameters included: (1) RR interval (average of 5 consecutive sinus RR intervals); (2) *P* wave duration (from the onset of *P* wave to the end of *P* wave); (3) QRS duration (from the onset of Q wave to the end of S wave); (4) QT interval (onset of Q wave to end of T wave, defined as the point where the T wave returns to the isoelectric line; the longest QT interval among 3 consecutive cardiac cycles was used to avoid underestimation,); (5) ICEB calculation: Defined as the ratio of QT interval to QRS duration (ICEB = QT/QRS), reflecting the balance between ventricular depolarization (QRS) and repolarization (QT).To eliminate the effect of heart rate, QTc was corrected by Bazett's formula (QTc = QT/√RR), ICEB was measured by the ratio of QTc/QRS.

### 2.3 Head-up tilt test protocol

All patients in study group were fasted for 4 h. HUTT was performed using SHUT-100 tilt-table system (Stanley Medical, Jiangsu, China; angle 0–90°, rise/fall ≤15 s, sampling 200 Hz). Baseline phase: Patients rested in the supine position for 5 min, during which continuous ECG and blood pressure (BP) were recorded. This phase ensured stabilization of autonomic tone before tilt initiation. Passive tilt phase: The table was tilted to 70° (head-up, feet-down) maintained for 20 min. ECG and BP were recorded continuously. Nitroglycerin (NTG) potentiation phase: If no syncope/presyncope occurred during the passive phase, patients remained tilted at 70°, and 0.3 mg nitroglycerin was administered sublingually. The test continued for an additional 15 min, with continuous monitoring of ECG and BP. The test was terminated immediately if: Syncope (loss of consciousness with loss of postural tone) or presyncope (symptoms including dizziness, lightheadedness, blurred vision, or nausea) occurred; Severe hypotension (systolic blood pressure < 80 mmHg) or bradycardia (heart rate < 35 beats/min) developed; Asystole > 5 s was detected; The patient requested termination due to intolerable symptoms (e.g., severe headache, chest pain).If no positive responses occurred during the passive and potentiation phases, the test was deemed negative (11). Positive results were classified as Type 1 (mixed), Type 2A (cardioinhibitory without asystole), Type 2B (cardioinhibitory with asystole), and Type 3 (vasodepressor) according to the new VASIS classification (12). Type 1 (mixed): Syncope associated with both ≥ 30 mmHg drop in systolic blood pressure and ≥ 20% drop in heart rate from baseline. Type 2A (cardioinhibitory without asystole): Heart rate<40 beats/min for > 10 s (or ≥ 50% drop from baseline) without asystole > 3 s, accompanied by syncope/presyncope. Type 2B (cardioinhibitory with asystole): Asystole > 3 s, accompanied by syncope/presyncope. Type 3 (vasodepressor): Syncope associated with ≥ 30 mmHg drop in systolic blood pressure, with no significant bradycardia (heart rate drop < 20% from baseline).

### 2.4 Statistical methods

Statistical analysis was performed using the SPSS 28.0 software. *P*-value of <0.05 was considered statistically significant. Prior to all analyses, normality tests (Shapiro–Wilk test and Q-Q plots) were conducted for continuous variables. The results confirmed that all continuous variables followed a normal distribution (all *P* > 0.05). Continuous variables are presented as mean ± standard deviation ($\bar{x}$ ± s), categorical variables are presented as numbers and percentages (%). Independent t-test was used for comparisons of continuous variables between groups, and chi-square test was for categorical variables. One-way analysis of variance (ANOVA) and chi-square test were used for comparisons of characteristics among different types, LSD and Tamhane's T2 and Bonferroni correction were applied for *post hoc* multiple comparisons. Multivariate linear regression analysis was used to explore the independent association between VVS and cardiac electrophysiological parameters.

## 3 Results

### 3.1 Demographic characteristics of the two groups

Demographic characteristics of two groups are presented in Table 1. There was no significant difference between the two groups in terms of age, body mass index, systolic blood pressure, and diastolic blood pressure (*P* > 0.05). However, the percentage of females was significantly higher among positive HUTT cases compared to the control group (*P* < 0.001).

**Table 1:** Comparison of demographic characteristics between two groups.

Clinical Data	Control Group (*n* = 201)	Study Group (*n* = 266)	*t*(*χ*^2^)	*P*
Age	52.22 ± 16.34	53.92 ± 17.14	−1.083	0.279
Female	96 (47.8)	174 (65.4)	14.627	<0.001
Body mass index, kg/m^2^	23.54 ± 2.19	23.61 ± 2.87	−0.284	0.777
Systolic blood pressure, mmHg	122.93 ± 10.53	123.08 ± 16.18	−0.117	0.907
Diastolic blood pressure, mmHg	78.49 ± 5.67	78.87 ± 9.41	−0.541	0.589

Values are mean ± SD or *n* (%).

### 3.2 Electrocardiographic characteristics of the two groups

Table 2 shows the ECG results of the two groups. RR interval, *P* wave and QRS duration were similar in two groups, with no statistical difference (*P* > 0.05). Compared to the control group, the QT interval (372.56 ± 31.00 ms vs. 402.16 ± 28.54 ms), QTc interval (406.57 ± 33.67 ms vs. 437.97 ± 29.51 ms), ICEB (3.96 ± 0.50 vs. 4.28 ± 0.46) and ICEBc (4.31 ± 0.55 vs. 4.66 ± 0.53) were lower in study group (*P* < 0.001, respectively).

**Table 2:** Comparison of electrocardiographic characteristics between two groups.

Variables	Control Group (*n* = 201)	Study Group (*n* = 266)	*t*	*P*	Cohen d	95% CI
RR interval, ms	851 ± 120	848 ± 118	0.271	0.787	−0.025	−0.208∼0.158
*P* duration, ms	95.97 ± 12.33	95.63 ± 12.16	0.292	0.771	−0.027	−0.210∼0.156
QRS duration, ms	94.68 ± 8.89	95.04 ± 9.41	−0.420	0.674	0.039	−0.144∼0.222
QT interval, ms	402.16 ± 28.54	372.56 ± 31.00	10.571	<0.001	−0.988	−1.181∼−0.794
QTc interval, ms	437.97 ± 29.51	406.57 ± 33.67	10.713	<0.001	−0.983	−1.176∼−0.789
ICEB	4.28 ± 0.46	3.96 ± 0.50	7.179	<0.001	−0.671	−0.859∼−0.482
ICEBc	4.66 ± 0.53	4.31 ± 0.55	6.824	<0.001	−0.638	−0.825∼−0.450

Values are mean ± SD. ICEB, The Index of Cardiac Electrophysiological Balance; ICEBc, corrected ICEB.

### 3.3 Electrocardiographic characteristics among different types of VVS patients and control group

**Figure 2:**
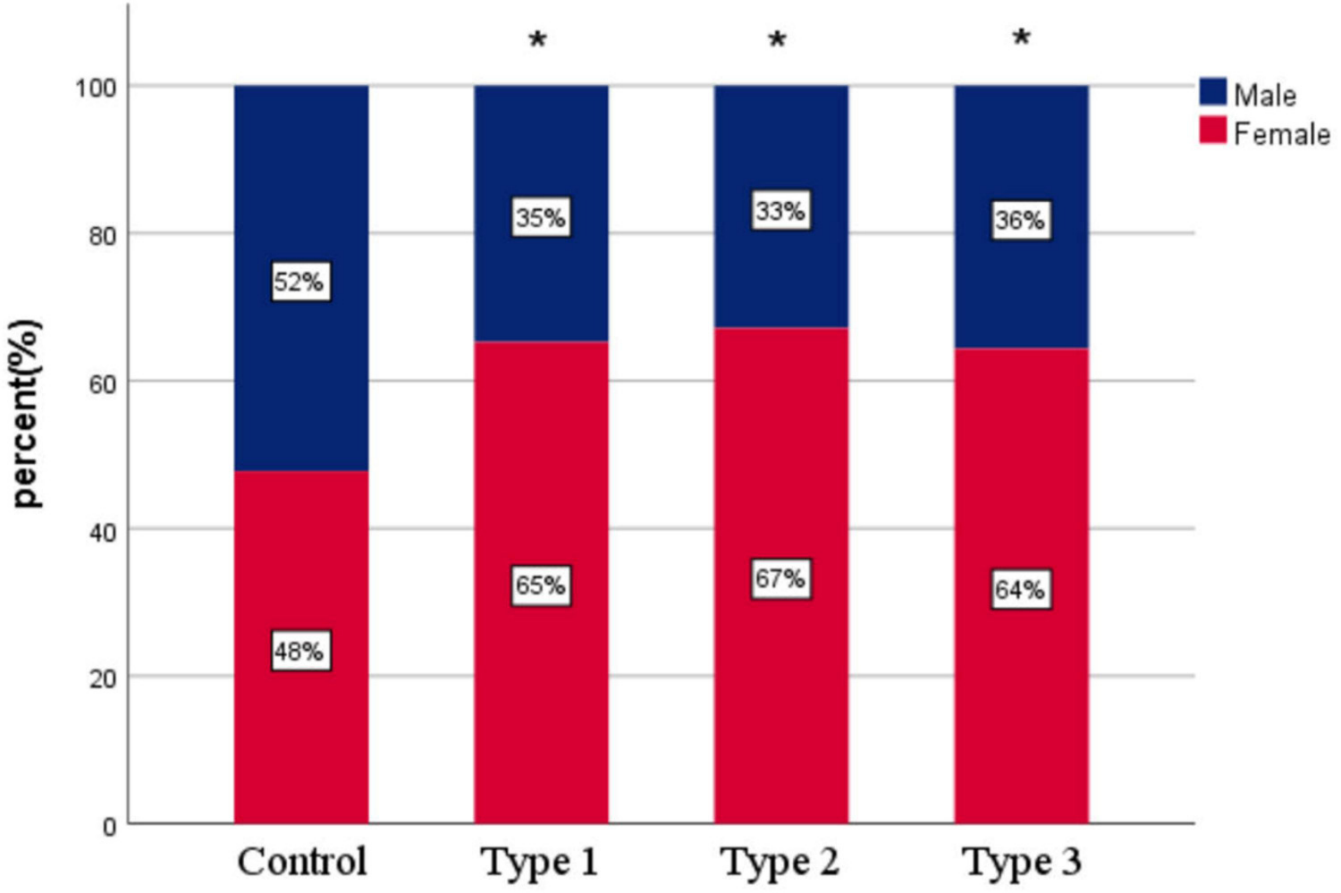
Proportion of females across different groups. Proportion of females across groups (control group and different types of Vasovagal Syncope). Female percentage ranged from 47.8% (control group) to 67.1% (Type 2 group). Overall *χ*^2^ test: *χ*^2^ = 14.760, *P* < 0.001; *post-hoc* pairwise comparisons with Bonferroni correction: adjusted *P* values < 0.008 (*α*/6) were considered significant. * Compared vs. control group, *P* < 0.008.

**Figure 3:**
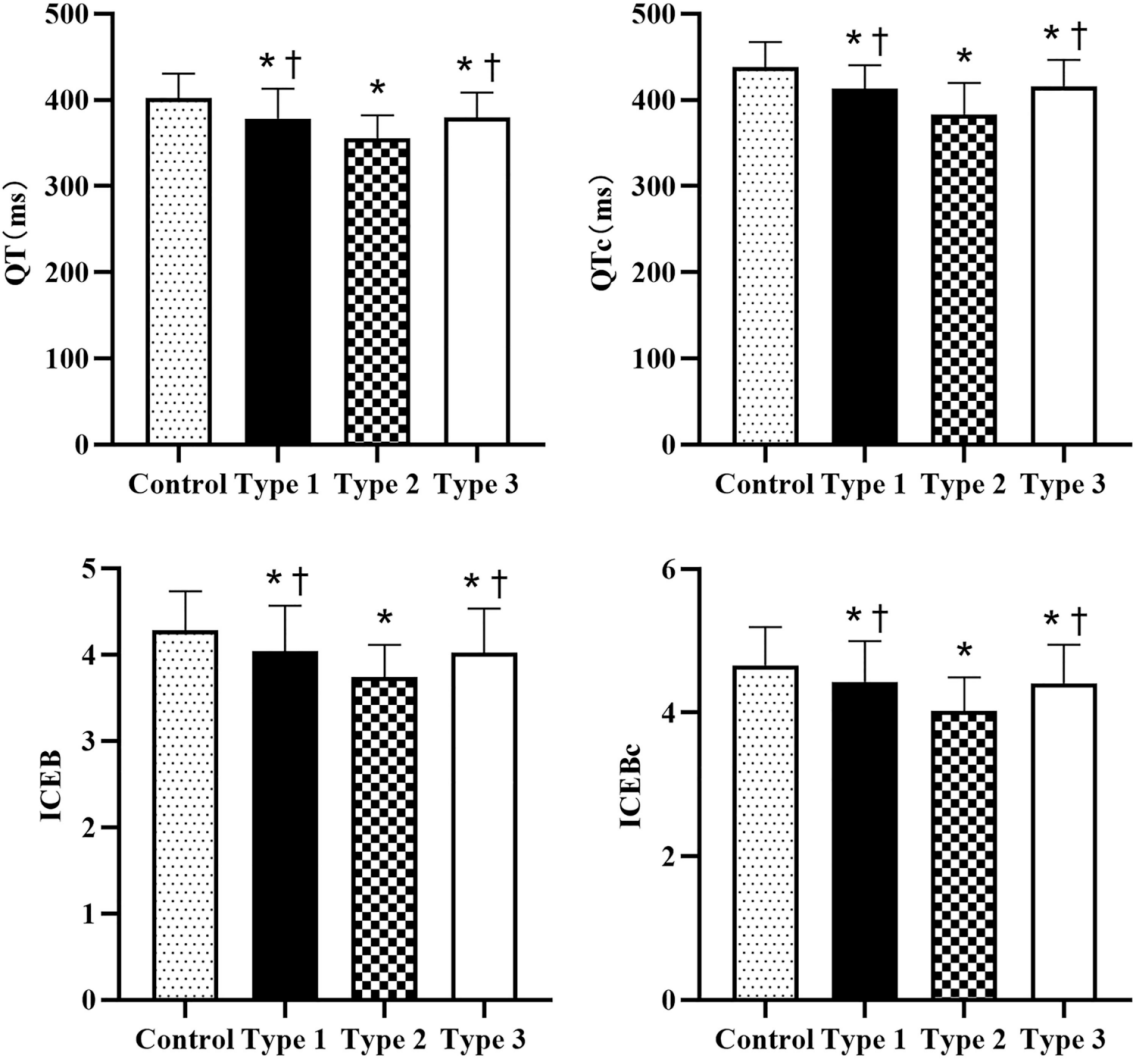
QT, QTc, ICEB, ICEBc across different groups. QT, QTc, ICEB, ICEBc across different groups. QT and QTc, the Index of Cardiac Electrophysiological Balance (ICEB) and corrected ICEB(ICEBc) in patients with different types of Vasovagal Syncope and control group: Data indicating that QT, QTc, ICEB, ICEBc are significantly decreased in positive Head-up tilt test (HUTT) patients, especially in Type 2 (cardioinhibitory). * Compared to control group, *P* < 0.05. ^†^ Compared to Type 2, *P* < 0.05.

To further investigate the electrocardiographic characteristics of VVS patients, we compared the clinical and ECG variables in all VVS patients. The total population in this cohort consisted of 266 positive patients, 95 patients in Type 1, 70 patients in Type 2, and 101 patients in Type 3. As shown in Table 3, the baseline characteristics were well-matched. Chi-square test and Bonferroni-corrected pairwise comparisons showed that proportion of female was higher in each positive type than in the control group (*P* < 0.008), with no significant difference among the subtypes (*P* > 0.008) (Figure 2). There was no statistical difference in RR interval, *P* wave and QRS duration in different types (*P* > 0.05), while the QT interval, QTc interval, ICEB and ICEBc were statistically altered (*P* < 0.001, respectively). These indicators were consistently lower in all positive groups than in the control group, with Type 2 showing the lowest level, even lower than those in Type 1 and Type 3, shown in Figure 3. In addition, Pearson's correlation coefficient was performed to explore the connection between the shortened ECG parameters and asystole duration in Type 2. Unfortunately, the results indicated that there was no significant correlation (*P* > 0.05, respectively, Table 4).

**Table 3:** Comparison of clinical and ECG variables among different groups.

Variables	Control (*n* = 201)	Type 1 (*n* = 95)	Type 2 (*n* = 70)	Type 3 (*n* = 101)	*F*(*χ*^2^)	*P*
Age	52.22 ± 16.34	54.71 ± 17.76	50.47 ± 14.36	55.58 ± 18.12	1.782	0.150
Female	96（47.8）	62（65.3）	47（67.1）	69（64.4）	14.760	0.002
Body mass index, kg/m2	23.54 ± 2.19	23.56 ± 2.76	23.57 ± 2.78	23.68 ± 3.05	0.055	0.983
Systolic blood pressure, mmHg	122.93 ± 10.53	125.31 ± 17.67	122.00 ± 11.19	121.72 ± 17.50	0.883	0.451
Diastolic blood pressure, mmHg	78.49 ± 5.67	79.37 ± 10.19	80.23 ± 6.91	77.47 ± 10.03	1.862	0.138
RR interval, ms	851 ± 120	837 ± 113	873 ± 112	842 ± 125	1.404	0.241
*P* duration, ms	95.97 ± 12.33	96.78 ± 10.75	93.20 ± 14.97	96.24 ± 11.08	0.991	0.398
QRS duration, ms	94.68 ± 8.89	94.34 ± 10.55	95.67 ± 7.13	95.26 ± 9.70	0.440	0.725
QT interval, ms	402.16 ± 28.54	376.79 ± 32.38	356.24 ± 26.18	379.88 ± 28.83	49.670	<0.001
QTc interval, ms	437.97 ± 29.51	413.20 ± 26.91	383.59 ± 36.20	416.25 ± 30.21	196.055	<0.001
ICEB	4.28 ± 0.46	4.04 ± 0.53	3.74 ± 0.37	4.03 ± 0.51	24.307	<0.001
ICEBc	4.67 ± 0.53	4.43 ± 0.56	4.03 ± 0.46	4.41 ± 0.54	26.014	<0.001

Values are mean ± SD or *n* (%). ICEB, The Index of Cardiac Electrophysiological Balance; ICEBc, corrected ICEB.

**Table 4:** Correlation analysis between QT, QTc, ICEB, ICEBc and asystole durations in type 2B patients with asystole.

Variables	*r*	*P*
QT	0.055	0.654
QTc	0.090	0.461
ICEB	0.085	0.486
ICEBc	0.123	0.312

ICEB, The Index of Cardiac Electrophysiological Balance; ICEBc, corrected ICEB.

### 3.4 Multivariate regression analysis of the association between VVS and electrophysiological parameters

To confirm the independent association between VVS and shortened ICEB, ICEBc, multivariate linear regression analysis was performed with age, sex, body mass index, systolic blood pressure, diastolic blood pressure as covariates. As shown in Table 5, after adjusting for age, sex, body mass index, systolic blood pressure, diastolic blood pressure, VVS patients still had significantly lower ICEB (*β*=-0.360, *P* < 0.001), ICEBc (*β* = −0.387, *P* < 0.001) compared with the control group. Among the covariates, only sex showed a significant independent association with ICEB (*β* = 0.191, *P* < 0.001) and ICEBc (*β* = 0.208, *P* < 0.001), while age, body mass index, systolic blood pressure, diastolic blood pressure had no significant effect on the above electrophysiological parameters (all *P* > 0.05). The adjusted R^2^ of the models ranged from 0.13 to 0.14.

**Table 5:** Association between VVS and electrocardiographic characteristics (adjusted for age, sex, body mass index, systolic blood pressure, diastolic blood pressure).

Dependent Variables	Independent Variables	*β*	Std. Error	*t*	*P*	95% CI	VIF
ICEB	Constant	4.240	0.283	14.969	0.000	3.683∼4.797	
	VVS	−0.360	0.045	−7.996	0.000	−0.448∼−0.271	1.035
	Age	0.003	0.001	1.836	0.067	0.000∼0.005	1.098
	Sex	0.191	0.045	4.228	0.000	0.102∼0.279	1.038
	Body mass index, kg/m^2^	−0.006	0.009	−0.743	0.458	−0.024∼0.011	1.067
	Systolic blood pressure, mmHg	0.001	0.002	0.583	0.560	−0.003∼0.005	1.499
	Diastolic blood pressure, mmHg	−0.002	0.003	−0.647	0.518	−0.009∼0.004	1.443
ICEBc	Constant	4.232	0.319	13.274	0.000	3.606∼4.859	
	VVS	−0.387	0.051	−7.643	0.000	−0.486∼−0.287	1.035
	Age	0.003	0.002	2.011	0.055	0.000∼0.006	1.098
	Sex	0.208	0.051	4.091	0.000	0.108∼0.307	1.038
	Body mass index, kg/m^2^	0.004	0.010	0.401	0.689	−0.015∼0.023	1.067
	Systolic blood pressure, mmHg	0.002	0.002	1.001	0.317	−0.002∼0.006	1.499
	Diastolic blood pressure, mmHg	−0.002	0.004	−0.640	0.522	−0.010∼0.005	1.443

*β*=unstandardized coefficient; VVS, Vasovagal Syncope; VIF, variance inflation factor; ICEB, The Index of Cardiac Electrophysiological Balance; ICEBc, corrected ICEB. Adjusted R^2^ for ICEB, ICEBc were 0.143, 0.134, respectively.

## 4 Discussion

Syncope is a frequently encountered clinical syndrome, with an estimated lifetime cumulative risk of approximately 40% individuals, though this figure varies by age and demographic characteristics. Among children and adolescents, the reported prevalence reaches 17.4%, and this trend escalates with advancing age (13). In those aged 80 years and above, the annual occurrence rate rises to 19.5 cases per 1,000 people (14). The underlying mechanisms of syncope are heterogeneous, encompassing neurally mediated reflexes-such as VVS and orthostatic hypotension. Although reflex syncope is often regarded as a benign disorder, recurrent and unpredictable episodes can be disabling, leading to substantial social consequences and economic burden. Large-scale studies consistently show that the incidence of VVS is significantly higher in females than in males, with a ratio of approximately 1.4–1.6:1, and this difference persists from adolescence through adulthood (15, 16). Our results also confirmed that female accounted for a significantly higher proportion in the syncope group.

The HUTT serves as a fundamental tool for diagnosing VVS and evaluating ANS. During the procedure, the patient is tilted to a 60°–80° upright position, while continuous monitoring of blood pressure and heart rate allows for assessment of autonomic responsiveness. VVS is categorized into three subtypes according to the positive responses to HUTT: cardioinhibitory, mixed, and vasodepressor. The first two are characterized by pronounced bradycardia or transient asystole, whereas the third manifests as a significant decline in systemic blood pressure, reflecting distinct autonomic profiles (11). It is reported that during HUTT, palpitations and tachycardia indicate heightened sympathetic activity. The subsequent sudden drop in blood pressure and heart rate results from sympathetic withdrawal and excessive parasympathetic drive, manifesting as flushing, sweating, dizziness (17, 18).

ANS dysfunction is one of the causes for changes in electrocardiogram. HRV is a common, noninvasive evaluation tool which reﬂecting both sympathetic and parasympathetic activity. HRV usually obtained through the measurement of 24-h ambulatory electrocardiogram. Nevertheless, previous HRV-based studies have reported inconsistent conclusions regarding autonomic changes in VVS (19–21). The inconsistent findings may partly stem from the lack of a unified, standardized approach to HRV assessment, including specific definitions, evaluation timing etc (22, 23). The QT interval duration represents the total electrical activity time of the ventricles from the onset of depolarization to the completion of repolarization, and is an important indicator for assessing ventricular repolarization time. QT interval includes QRS duration, the JT-peak (JTp) interval, and the T-peak-to-T-end (Tp–Te) interval. In recent years, a more sensitive electrophysiological indicator—ICEB, which was given by the QT/QRS, has been proposed as a manifestation of the balance between cardiac repolarization and depolarization (1). Just like corrected QT by heart rate, ICEB is also corrected by heart rate. Both excessively high or low ICEB values may lead to malignant arrhythmias. ICEB is not merely a “static” electrophysiological parameter, rather, it can be dynamically regulated by the ANS. Yucetas et al. reported that ICEB was significantly higher in patients with aneurysmal Subarachnoid Hemorrhage (SAH), which may be positively linked to autonomic dysfunction and heightened sympathetic activity (2). Studies on dapagliflozin in HFrEF showed that, while improving sympathetic/parasympathetic balance and reducing repolarization dispersion, dapagliflozin lowers ICEB by 17.9%. ICEB was positively correlated with NT-proBNP, and the authors held that “autonomic modulation” as one of the three principal mechanisms underlying the ICEB reduction (24). In our study, QT, QTc, ICEB and ICEBc were significantly lower in VVS cases than in healthy cases in the basic lying position. Furthermore, among the three subtypes, the value of type 2 is even lower. This shortening of ICEB and ICEBc is mainly derived from shortened QT and QTc interval in VVS, as the QRS duration remained unchanged compared with the control group. Although the reason remains unknown, we speculated that it is related to impaired cardiac autonomic function, which involve dysregulation of both the sympathetic and parasympathetic systems. Our findings are consistent with previous studies, that is high ICEB indicates sympathetic predominance (2), conversely, lower ICEB suggests parasympathetic predominance. There is controversy regarding the basal autonomic function status in patients with VVS. Some studies have suggested that there is no significant abnormality in the autonomic nerve function of patients with VVS during the asymptomatic phase: Baran et al. and Nussinovitch et al. conducted HRV analysis on VVS negative and positive groups, and found no difference in basal autonomic tone between the two groups (25, 26). Inconsistently, Sharad et al. revealed that patients with reflex syncope exhibit a distinctive ambulatory blood pressure profile during resting conditions, reflecting underlying autonomic nervous system dysfunction (27). Similar to the findings of Sharad et al., we observed that patients with VVS showed lower baseline ICEB, which suggests the existence of chronic autonomic dysfunction with vagal predominance. However, we did not verify HRV data. Validation of this phenomenon in subsequent studies and exploration of its underlying mechanisms are urgently needed for our next research step. Additionally, the core finding of this study was confirmed by multivariate linear regression analysis: after adjusting for potential confounders (age, sex, body mass index, systolic blood pressure, diastolic blood pressure), VVS patients still exhibited significantly shortened ICEB, ICEBc compared with healthy controls. This result indicates that the electrophysiological abnormalities observed in VVS patients are independently associated with VVS itself, rather than being mediated by age, sex, or body mass index, systolic blood pressure, diastolic blood pressure. Among the included variables, VVS status had the largest absolute standardized regression coefficient (*β* = −0.360 to −0.387), indicating that VVS is one of the most important factors contributing to the shortening of ICEB and ICEBc. This finding reinforces the clinical significance of ICEB as a potential electrophysiological marker for VVS, as its association with VVS is robust to adjustment for common confounding factors. Regarding the covariates, sex showed independent association with ICEB (*β* = 0.191, *P* < 0.001) and ICEBc (*β* = 0.208, *P* < 0.001), which aligns with the higher prevalence of VVS in females observed in our study and previous reports. This suggests that female may be a minor contributing factor to electrophysiological imbalance, possibly related to sex-specific differences in autonomic regulation or hormonal profiles.

Notably, we also investigated the relationship between QT, QTc, ICEB and ICEBc with the duration of cardiac arrest in patients with Type 2 of VVS. Unfortunately, there was no strong correlation. This may be attributed to the distinct pathological mechanisms underlying these two indices: ICEB reflects the intrinsic baseline electrophysiological balance of ventricular depolarization-repolarization, which is a pre-existing trait mediated by chronic autonomic dysfunction (elevated vagal tone). In contrast, asystole duration is a dynamic response to acute orthostatic stress, determined by the intensity/duration of paroxysmal vagal activation, individual neuroreflex sensitivity, and hemodynamic changes during HUTT. The static baseline electrophysiological abnormality (ICEB shortening) does not directly correlate with the dynamic severity of acute neurohemodynamic reflex, leading to the lack of significant correlation.

Conventional diagnosis of VVS depends on HUTT. This study suggests shortened baseline ICEB may act as a prescreening biomarker for VVS. In patients with unexplained syncope, ICEB calculated from baseline ECG is simple, noninvasive and low-cost, and can serve as an initial risk stratification tool. Additionally, ICEB was lowest in Type 2 (cardioinhibitory) VVS, indicating more severe baseline electrophysiological instability in this subtype. This may help identify high-risk cardioinhibitory patients. These findings not only enrich our understanding of ICEB as a bidirectional marker of autonomic balance but also provide a simple, noninvasive tool for VVS evaluation, opening new perspectives for baseline electrophysiological profiling and intervention monitoring in VVS.

## 5 Limitations

There are some limitations. First, the single-center, retrospective design and moderate sample size inherently limit the generalizability of our findings. The subtle nuances of cardiac electrophysiological balance may vary across diverse populations and clinical settings, necessitating validation through large-scale, multicenter prospective studies to unravel its full clinical relevance. Second, population-based normative ranges for ICEB and ICEBc remain elusive, as no unified reference standards have been established to date. Third, while we speculate that ICEB shortening is linked to autonomic dysfunction, direct evidence from HRV or other autonomic function assessments is absent in this study. The intricate interplay between ventricular electrophysiological balance and autonomic regulation-whether it involves a primary neural imbalance driving electrical instability or vice versa-remains an uncharted territory that warrants deeper mechanistic investigation.

## 6 Future directions

1. Given the single-center and retrospective nature of the current study, future research should adopt a multicenter, prospective design with a larger sample size. This will help verify the stability and generalizability of the association between ICEB/ICEBc and VVS across diverse populations, regions, and clinical settings.
2. Currently, there are no unified population-based normative ranges for ICEB and ICEBc. Future research should focus on constructing standardized reference values for ICEB and ICEBc across different age groups, genders, and physiological states. This will enable clinicians to accurately assess whether a patient's ICEB/ICEBc falls within the pathological range, thereby enhancing the clinical utility of these indices in the diagnosis of VVS.
3. The current study speculated that ICEB shortening in VVS patients is associated with ANS dysfunction, but more direct evidence (e.g., HRV, sympathetic/parasympathetic activity measurements) was lacking. Future studies should integrate multiple assessment tools for autonomic function, such as 24-h ambulatory electrocardiography (to measure HRV), skin sympathetic nerve activity monitoring, and pupillary reflex tests. Correlating these data with ICEB/ICEBc values will enable researchers to clarify whether ANS dysfunction (e.g., abnormal vagal-sympathetic balance) directly drives ventricular depolarization-repolarization imbalance, and identify the specific neural regulatory pathways involved.

## 7 Conclusions

In our study, the QT, QTc, ICEB and ICEBc in VVS patients were investigated. Results show that QT, QTc, ICEB and ICEBc were significantly shortened in VVS at baseline, particularly in Type 2 patients. While this change showed no significant positive correlation with the duration of cardiac arrest in patients with Type 2 VVS. Nevertheless, more in-depth research is still needed.

## Data Availability

The raw data supporting the conclusions of this article will be made available by the authors, without undue reservation.
